# Focus on overview of innovative materials for energy

**DOI:** 10.1080/14686996.2017.1379729

**Published:** 2017-09-28

**Authors:** Fabien Grasset, Laurent Pic

**Affiliations:** ^a^ Laboratory for Innovative Key Materials and Structures, UMI 3629 LINK, NIMS-Saint Gobain-CNRS, National Institute of Material Science, Tsukuba, Japan; ^b^ Ambassador of France to Japan

Innovative materials for energy play an important role in major fields of science and technology, including life and environmental sciences and many interdisciplinary fields. This focus issue contains several reviews and primary research articles devoted to materials for energy conversion (photovoltaic, lighting, solid oxide fuel cells, etc.) and energy harvesting applications. This focus issue was organized within a collaboration between Japan and France. Half of its articles originate from joint research structures, such as the International Joint Research Unit (UMI 3757 ELytMax, UMI 3629 LINK) and the Associated International Laboratory (LIA NextPV). This issue also features works carried out in close partnership between French and Japanese research teams in areas related to materials for energy. It clearly demonstrates the dynamic exchange in this field between these two countries, from basic to applied science.

This Foreword includes the encouraging words of Laurent Pic, the Ambassador of France to Japan. We would like to thank him and the Embassy of France in Japan for their support. We are also grateful to all the involved authors and reviewers, as without their help this focus issue would not have been published on time.

## Introduction by Laurent Pic, Ambassador of France to Japan 

In the context of the Paris Agreement on Climate Change, France and Japan are seeking an optimal energy mix that combines conventional and alternative pathways and is able to meet the demand for energy while protecting the environment. Secure, competitive, and sustainable, the optimal energy mix requires significant research and innovation efforts to ensure the improvement of thermal energy technologies and the development, integration, and storage of reliable renewable energies.

Striving for tomorrow’s energy mix thus requires technologies using increasingly efficient materials that are sustainable and usable under potentially extreme conditions, while improving the reliability and safety of structures and facilities. The success of research and development activities, as measured by their ability to provide sustainable technology solutions, relies on the design of new materials adapted to environmental constraints.

In order to achieve the optimal energy mix, France and Japan have thus placed great emphasis on materials for energy, a field in which both countries boast world-class expertise driven by flagship research institutions and companies. France and Japan have also developed extensive cooperation in this field, as illustrated by the establishment of three major joint laboratories within the French National Center for Scientific Research (CNRS). This focus issue, dedicated to the subject of innovative energy materials, features high-quality contributions from each of these laboratories.

The Embassy of France in Japan strongly supports French–Japanese cooperation on materials for energy through numerous scientific seminars and events such as the French–Japanese Seminar on Materials for Energy, organized in 2014 in collaboration with the Science Council of Japan. The France–Japan Innovation Year, launched in 2015 by the prime ministers of both countries, also provided a framework for several events, including a French–Japanese Symposium on Green Hydrogen Production and Storage held in Osaka in 2016.

This focus issue shows the diversity and significance of French–Japanese cooperation in the field of materials for energy and provides a wonderful opportunity for me to reaffirm the desire of the Embassy of France in Japan to fully support this cooperation. It is my hope that it will continue to grow and become a breeding ground for tomorrow’s sustainable energy technologies.

Fabien Grasset *Laboratory for Innovative Key Materials and Structures, UMI 3629 LINK, NIMS-Saint Gobain-CNRS, National Institute for Material Science, Tsukuba, Japan* fabien.grasset@univ-rennes1.fr Laurent Pic Ambassador of France to Japan Ambassade de France, Minami-Azabu, Minato-ku, Tokyo, Japan

